# RAPPORT: An Open-Source R Shiny Application for Breeding Program Management with Integrated Pedigree-Based Genetic Monitoring

**DOI:** 10.3390/ani16213491

**Published:** 2026-11-04

**Authors:** Birgit Deboutte, Luc Peelman, Bart J. G. Broeckx

**Affiliations:** Department of Veterinary and Biosciences, Faculty of Veterinary Medicine, Ghent University, 9820 Merelbeke-Melle, Belgium; birgit.deboutte@ugent.be (B.D.); luc.peelman@ugent.be (L.P.)

**Keywords:** breeding management, inbreeding, pedigree analysis, mean kinship, genetic diversity, assistance dogs, effective population size

## Abstract

Managers of closed or semi-closed animal breeding programs must balance production targets with the preservation of genetic diversity, yet they often lack training in population genetics and access to integrated, user-friendly tools. RAPPORT (Relatedness Analysis and Population Planning, Organisation, Reporting and Tracking) is a freely available, open-source R Shiny application that combines pedigree-based genetic monitoring with operational breeding management in a single no-code interface. The application computes inbreeding coefficients and pairwise kinship, estimates rate of inbreeding (*ΔF*) and effective population size (*N_e_*), and visualises inbreeding trends with forward projections. Production planning tools include dam scheduling, litter projections, and phenotype-integrated breeder selection with mean kinship ranking. Mating recommendations are filtered by a per-mating inbreeding increase threshold and user-defined health criteria. Pedigree and phenotypic data are uploaded in Excel format, and any user-defined traits are automatically incorporated into downstream analyses. Using a test case with anonymised data from a Belgian assistance dog breeding program (852 pedigree entries, 65 litters), the numerical correctness of RAPPORT’s pedigree-based genetic metrics was compared and confirmed against established R packages, and RAPPORT’s capacity to identify a production shortfall and support breeding decisions integrating genetic and phenotypic criteria was illustrated. RAPPORT makes population-level genetic management accessible to non-specialist breeding program managers.

## Simple Summary

Breeding programs, such as for assistance dogs, must produce enough healthy animals to meet demand while also keeping the population genetically diverse. When these animals become too closely related, their offspring are more likely to inherit health problems. However, many program managers lack formal training in genetics and struggle with software that is either too technical or only addresses part of the problem. We developed RAPPORT, a free, open-source computer application that brings together genetic monitoring and day-to-day breeding management in one easy-to-use interface. Users simply upload their records in spreadsheet format, and the application automatically calculates how related the animals are, tracks trends in genetic diversity over time, and projects future changes. It also helps plan which animals to breed and when, it suggests pairings that keep relatedness low, and incorporates health and trait information into those recommendations. We verified RAPPORT’s genetic calculations against independent tools and found them to be identical. A test case using data from a Belgian assistance dog breeding program illustrated that RAPPORT could identify upcoming production shortfalls, and support informed breeding decisions. By making these tools accessible to non-specialists, RAPPORT helps breeding programs produce healthier animals while safeguarding long-term population health.

## 1. Introduction

Managing closed or semi-closed animal breeding programs can be challenging. On the one hand, the manager must handle the practical side of running a breeding program: deciding how many animals to breed each year, which dams are available and for how long, how many new breeders to recruit, and whether production is on track. On the other hand, they need to track the genetic health of the population. As the pool of available partners narrows and the same bloodlines remain across generations, inbreeding might accumulate, the effective population size (*N_e_*) shrinks, and genetic diversity decreases. These changes happen gradually, so that no single breeding decision looks alarming. The challenge is not that these problems are hard to solve; it is that they are often not noticed before it is too late. The outcome may be dramatic: increasingly inbred populations at risk of inbreeding depression, which may manifest as rising disease prevalence, reduced fertility, and smaller litter sizes [1,2,3,4,5,6,7].

Practically, the managers responsible for the current breeding programs are, in most cases, not quantitative geneticists and have little formal background in population genetics or quantitative methods. There is a knowledge gap that has been formally documented in conservation programs [8], which is also present in pet breeding contexts [9,10]. Chen et al. [11] noted that working dog breeders struggle to apply the tools currently available, hindered by the need for dedicated data management and statistical expertise. There thus seems to be a structural mismatch between the tools that exist and the people who need to use them. Addressing this requires software that is genuinely accessible to non-specialists: one that handles the underlying computations transparently, presents results in plain language, and integrates the genetic and operational dimensions of program management into a single workflow. Table 1 contains more details on the practical characteristics that seem to be necessary to fulfil this.

A detailed evaluation of the software that is currently available for breeding management illustrates that the software can be broadly divided into two groups. On one hand, simulation frameworks such as MoBPS [12] and AlphaSimR [13] offer powerful environments for the theoretical design and optimisation of breeding strategies, but operate on virtual, simulated populations rather than real studbook data.

The other group comprises the tools designed for the management of real, existing populations, which can be further subdivided into three categories. At the research end, tools such as optiSel [14] are powerful and sophisticated frameworks for optimum contribution selection and breeding program simulations, though they are primarily designed for geneticists evaluating large commercial or research populations, and their full functionality requires either knowledge of R scripting or familiarity with complex simulation parameters. Retriever and Pointer [15] and POPREP [16] were developed specifically for small, closed populations and are more accessible, offering pedigree-based *N_e_* estimates and kinship-guided mating recommendations via graphical interfaces; they are focused on the genetic monitoring and mating recommendation layer, rather than demographic planning, phenotypic selection criteria, or breeder selection tools. At the consumer end, tools such as ZooEasy [17] and The Breeder’s Standard [18] are widely used by hobby and small-scale breeders for pedigree management and inbreeding calculation, with a focus on individual animal records rather than population-level monitoring, phenotype-integrated selection workflows, or operational production planning.

Finally, at the practical management end, BCAS [19] and The International Working Dog Registry (IWDR) [11,20,21] represent more integrated approaches. BCAS combines mean kinship-based breeder selection with mating recommendations and has been successfully applied in endangered breed management, though its scope does not extend to operational planning tools such as litter projections or production capacity management. IWDR represents the most integrated approach currently available, combining estimated breeding value (EBV) calculation, inbreeding management, and phenotypic record-keeping in a single collaborative database. While primarily focused on genetic selection and breeding decisions, it currently does not include operational planning tools such as dam scheduling, litter projections, or production capacity management. It is also designed specifically for working dog programs and relies on collaborative data sharing across organisations for its genetic selection tools to function optimally. Breed registries, conservation programs, and smaller independent breeding organisations, the populations most vulnerable to the genetic risks described above, fall largely outside its current scope. Table 1 summarizes the existing tools.

Collectively, these tools have made important contributions to genetic management in animal breeding programs, and RAPPORT builds on this foundation by aiming to integrate genetic monitoring with operational breeding management in a single interface accessible to non-specialist users. Based on the detailed comparison of the software currently available (Table 1), there indeed seems to be room for a tool that fulfils the aforementioned criteria. Our goal was to develop software with a graphical user interface, suited for these kinds of analyses. After the development, we performed a practical evaluation based on real-world data.

**Table 1:** Comparative overview of breeding program management tools.

	optiSel	Retriever & Pointer	POPREP	ZooEasy	The Breeder’s Standard	BCAS	IWDR	RAPPORT
DIVERSITY METRICS								
Inbreeding coefficients	yes	yes	yes	yes	yes	yes	yes	yes
Effective population size (*N_e_*)	yes	yes	yes	no	no	yes	yes	yes
Mean kinship monitoring	yes	yes	yes	yes	no	yes	no	yes
Mean kinship-based breeder selection *	no	no	no	no	no	yes	no	yes
DEMOGRAPHIC METRICS								
Generation interval & litter statistics **	no	yes	yes	no	no	partially	partially ^1^	yes
Population overview ***	no	yes	yes	no	no	yes	partially ^2^	yes
Individual trait monitoring	no	no	no	yes	yes	yes	yes	yes
Population-level trait monitoring	no	no	no	no	no	no	yes	yes
BREEDING MANAGEMENT								
Production planning & projections	no	no	no	no	no	no	partially ^3^	yes
Phenotypic breeder selection criteria	no	no	no	no	no	no	yes	yes
EBV calculation	no	no	no	no	no	no	yes	no
EBV integration ****	yes	no	no	no	no	no	yes	yes
Ranked mating recommendations	yes	partially ^4^	no	partially ^5^	partially ^5^	yes	yes	yes
ACCESSIBILITY								
Interactive interface (no coding required)	no	partially ^6^	yes	partially ^6^	yes	yes	yes	yes
Works with user-supplied data files	yes	yes	yes	no	no	no	no	yes
Free to use	yes	yes	yes	no	no	no	no	yes

* Breeding candidates are ranked by individual mean kinship following Oliehoek et al. [22]. ** Includes mean litter size, mean litter interval, and mean number of litters per dam per year. *** Summary of population-level demographic and genetic parameters over time. **** For RAPPORT and optiSel, externally calculated estimated breeding values (EBVs) can be imported as traits. For IWDR, EBVs are calculated within the platform itself. ^1^ IWDR records reproductive data but does not provide litter-level production statistics. ^2^ IWDR is focused on individual animal records rather than population-level overviews. ^3^ IWDR records detailed reproductive data per animal, including estrus cycles, whelping records, and litter history, but does not provide production planning tools or projections for population-level output targets. ^4^ Retriever/Pointer can simulate mating restrictions based on kinship thresholds but does not produce an individually ranked candidate list. ^5^ ZooEasy and The Breeder’s Standard support test matings with coefficient of inbreeding prediction but without ranking. ^6^ Retriever/Pointer and POPREP provide a graphical interface for data input but generate static output; results cannot be explored interactively within the application.

## 2. Materials and Methods

### 2.1. The Software Overview

RAPPORT (Relatedness Analysis and Population Planning, Organisation, Reporting and Tracking) is freely available at https://github.com/BirgitDeboutte/RAPPORT (accessed on 12 October 2026) under a GPL-3.0 license. All modules share a single reactive data object that is passed across the application. This ensures that any change to the data, for example, designating a new breeder or appending an external candidate, is immediately reflected in all downstream computations and displays. Key package dependencies include shiny (v1.13.0), bslib (v0.10.0), readxl (v1.4.5), DT (v0.34.0), plotly (v4.12.0), dplyr (v1.2.1), pedigree (v1.4.2) and kinship2 (v1.9.6.2). RAPPORT has been built in R version 4.5.3.

### 2.2. Input Data and Preprocessing

Two Excel (.xlsx) input files are accepted: a pedigree file (animal ID, dam ID, sire ID, sex) and a data file (animal ID, sex, date of birth; optional columns for own-population flag, breeding stock designation, and phenotypic traits). Animal IDs must match exactly between files. On submission, both files pass through a multi-step quality control and preprocessing pipeline before any downstream computation. For the data file, dates are parsed robustly with fallback format detection, and character columns containing European decimal notation are converted to numeric. Columns with ambiguous content (a mix of numeric and text values) are flagged to the user. Missing required columns and column-mapping collisions (the same column assigned to multiple roles) are validated before processing continues. For the pedigree file, the following checks are applied automatically upon submission: whitespace trimming on all identifier columns; normalisation of missing-value synonyms (empty strings, “0”, “unknown”, “NA”, and “.” are all converted to NA); single-parent nulling, where animals with only one recorded parent have both parents set to unknown, as the tabular method requires either both parents or neither; detection and removal of duplicate animal identifiers, retaining the first occurrence; sex-role mismatch detection, where animals appearing in the dam column but recorded as male (or vice versa) are flagged and their sex is auto-corrected to match their parental role; dam/sire column swap detection, triggered when more than 50% of identifiable dams are male or more than 50% of sires are female, in which case submission is blocked with an explanatory error; self-parentage detection, where animals listed as their own dam or sire have the offending parent set to unknown; phantom parent insertion, where individuals referenced as parents but absent from the identifier column are added as founder rows; and topological sorting, ensuring all parents precede their offspring in the processed pedigree. The overlap between data file and pedigree file identifiers is checked, and unmatched individuals are reported. All findings are summarised in a colour-coded quality control report panel displayed immediately after pedigree submission, allowing the user to review and, if necessary, correct the source files before proceeding to analysis. There is no software-imposed limit on pedigree depth; computation proceeds from founders (animals with no recorded parents) through to the most recent generation, and the depth of the pedigree is captured by the equivalent complete generations (ECG) statistic at the population level.

### 2.3. Analytical Scopes

RAPPORT unites diversity metrics, demographic metrics, and breeding management projections in a single application, encompassing inbreeding and pairwise kinship coefficients, individual generation depth and equivalent complete generations, effective population size, generation interval and litter statistics, population growth projections, mean kinship, breeder selection, and ranked mating.

Inbreeding coefficients and pairwise kinship are always derived from the full pedigree file, including all historical ancestors and animals outside the own population. Population-level summary statistics (equivalent complete generations (ECG), Δ*F*, and *N_e_*) are computed over the reference population when a data file is provided, or over the full pedigree otherwise. Mean litter size, generation interval, litter interval, active dam and sire counts, and all management projections are computed exclusively from the own-population subset of the data file, reflecting the operational reality of the program being managed.

### 2.4. Inbreeding and Pairwise Kinship Coefficients

Individual inbreeding coefficients and pairwise kinship coefficients are computed from the full sorted pedigree using Henderson’s tabular method [23]. The kinship coefficient *k_ij_* between individuals *i* and *j* is defined as the probability that a randomly drawn allele from *i* and a randomly drawn allele from *j* are identical by descent (IBD). For these calculations, animals with only one known parent have both parents set to unknown; founders are added and the pedigree is topologically sorted so all parents precede their offspring. Working through individuals in topological order, the complete *n* × *n* kinship matrix *K* is built row by row: for each new individual *i*, its kinship with any earlier individual *j* is simply the average of its parents’ kinships with *j* (values already computed in previous rows). Because kinship is symmetric (*k_ij_* = *k_ji_*), only the upper or lower triangle needs to be computed explicitly. The restriction *j* < *i* only traverses the lower triangle, with the upper triangle following by symmetry. The kinship *k_ij_* between any two individuals *i* and *j* (*j* < *i*) with *s_i_* and *d_i_* the sire and dam of individual *i*, is thus computed as:

$$k_{\mathrm{ij}}=\;k_{\mathrm{ji}}=\frac{\left(k_{s_{i},j}+\;k_{d_{i},\;j}\right)}{2}$$

$k_{s_{i},j}$ and $k_{d_{i},\;j}$ are the kinship of the sire and dam of individual *i*, with individual *j*, respectively. Here, the kinship contribution of an unknown parent is zero. On the diagonal, the kinship of individual *i* follows from the kinship between the two parents:

$$k_{\mathrm{ii}}=\frac{\left(1\;+\;k_{s_{i},\;d_{i}}\right)}{2}$$

where $k_{s_{i},\;d_{i}}=\;0$ if either parent is unknown (founder assumption). Because every new value in the kinship matrix is calculated reusing what is already in the matrix, no pedigree traversal is repeated.

The inbreeding coefficient *F_i_* for individual *i* is recovered from the diagonal as:

*F_i_* = 2*k_ii_* − 1

**Figure 1:**
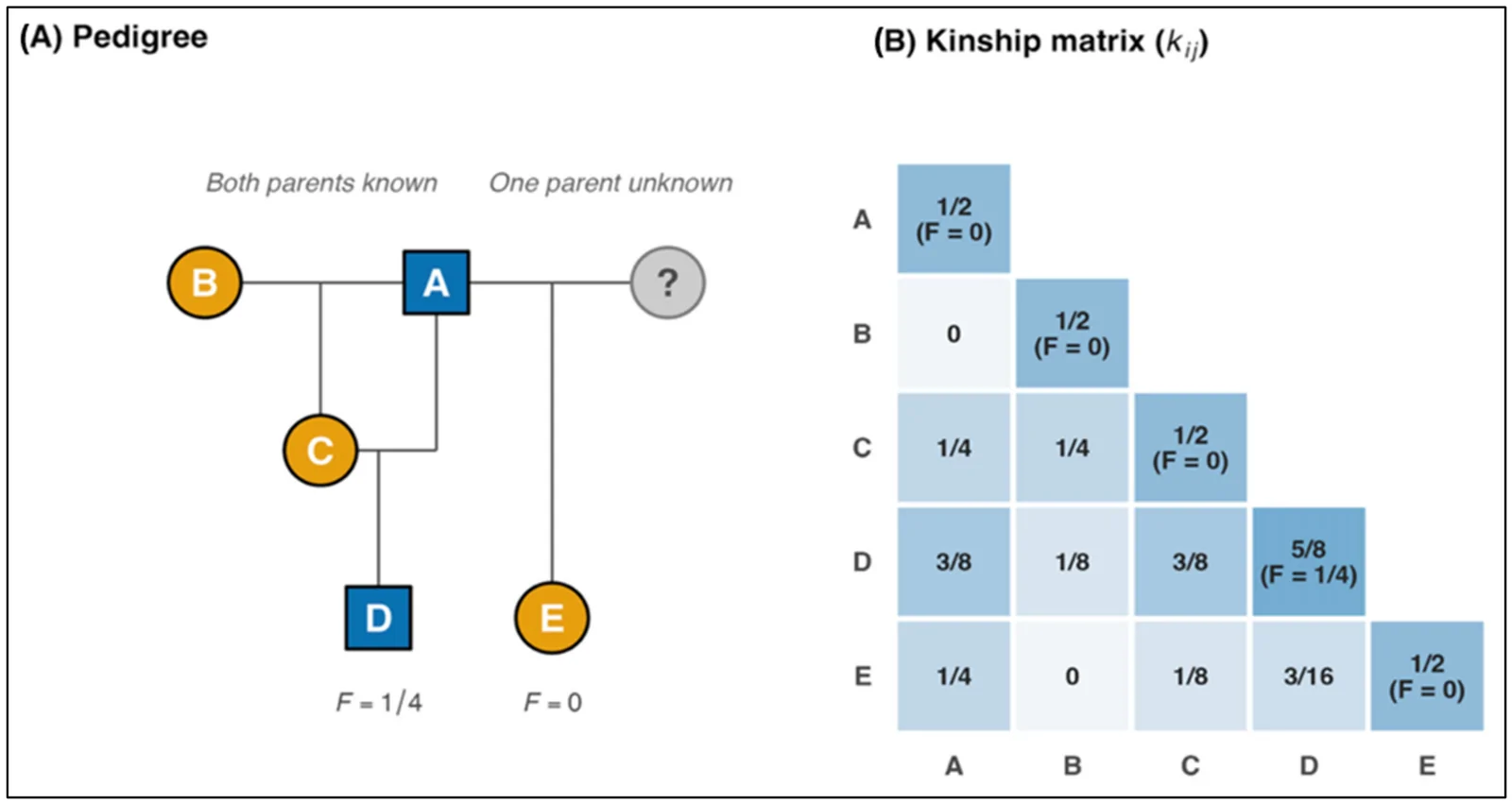
An example of the tabular method for computing kinship coefficients. (**A**) A five-individual pedigree with two unrelated founders (A, B) and one unknown parent (?), illustrating two different matings. (**B**) The resulting lower-triangular kinship matrix, with inbreeding coefficients (F) on the diagonal.

Equivalently, *F_i_* equals the kinship between the individual’s parents: $F_{i}=\;k_{s_{i},\;d_{i}}$ (Figure 1). This formula is used in the Ranked Mating module (see below) to compute the expected inbreeding coefficient of prospective offspring. All individuals without recorded parents are treated as non-inbred, unrelated founders. Inbreeding estimates are therefore conditional on pedigree depth: when pedigree incompleteness increases, the inbreeding estimates become increasingly more underestimated.

### 2.5. Individual Generation Depth and ECG

The generation depth *t_i_* of individual *i* is the sum of known ancestor contributions weighted by their generation distance [24]. It is computed by a single forward loop through the sorted pedigree:

$$t_{i}=\frac{\left(1\;+\;t_{s_{i}}\right)}{2}+\frac{\left(1\;+\;t_{d_{i}}\right)}{2}$$

where $t_{s_{i}}$ and $t_{d_{i}}$ are the generation depths of the sire and dam of individual *i*, respectively; unknown parents contribute 0, and founders have *t* = 0 by definition. The ECG is defined as $\bar{t_{i}}$ across the animals in the reference population and serves as an indicator of overall pedigree completeness.

### 2.6. Effective Population Size

The *N_e_* is estimated following González-Recio et al. [25], as described in Gutiérrez et al. [26]. For each animal *i* with *t_i_* > 0 (i.e., animals with at least some known ancestry, excluding the founders), the individual increase in inbreeding *ΔF_i_* is:

$$\Delta F_{i}=\;1\;-\;\sqrt[{t_{i}}]{\left(1\;-\;F_{i}\right)}$$

These individual estimates are averaged over the reference population when a data file is provided, or over the full pedigree otherwise, to estimate the average rate of inbreeding $\bar{\Delta F}$:

$$\bar{\Delta F}\;=\frac{1}{N}\sum_{i=1}^{N}\Delta F_{i}\;$$

The effective population size is then:

$$N_{e}=\;\frac{1}{2\bar{\Delta F}}$$

A minimum of 20 valid individuals with known ancestry is required for *N_e_* estimation, as fewer individuals yield individual *ΔF_i_* estimates with variance too large for the population-level average to be informative; shallower pedigrees return *N_e_* as unavailable. Interpretation thresholds throughout the application follow the revised minimum viable population standards [27,28]: *N_e_* > 200 is considered adequate (with *N_e_* > 200 defined as a conservative management target to provide a safety margin above the Frankham et al. (2014) minimum threshold, reflecting the tendency for *N_e_* estimates from pedigrees to be upward-biased); *N_e_* between 100 and 200 is acceptable; *N_e_* between 50 and 100 indicates a population at risk of diversity loss; *N_e_* < 50 indicates a critical lack of diversity.

The ‘Trends’ module additionally visualises mean inbreeding per birth year with a weighted linear regression fit, a 95% confidence band, and a five-year forward projection. A reference line at 1% per generation, inspired by the *N_e_* < 50 critical threshold applied throughout the application, is overlaid as a practical ceiling on inbreeding accumulation; it is converted to a per-year slope using the estimated generation interval L (see below). The slope of the cohort trend and the population-level average rate of inbreeding ($\bar{\Delta F})$ measure related but distinct quantities: recent rate of change versus lifetime accumulation averaged across animals (see above) and can differ when inbreeding is accelerating or slowing. Both are displayed on the plot to make any divergence immediately visible.

### 2.7. Generation Interval and Litter Statistics

A litter is defined as all offspring sharing the same date of birth, dam, and sire. The generation interval *L* of the population is computed as the litter-size-weighted mean age of dams at birth of offspring:

$$L\;=\frac{\sum_{k=1}^{K}\bar{a_{k}}\;\cdot \;\bar{n_{k}}}{\sum_{k=1}^{K}\bar{n_{k}}}$$

where $\bar{a_{k}}$ and $\bar{n_{k}}$ are the mean dam age and mean litter size at the *k*-th litter averaged across all dams that produced at least *k* litters, and *K* is the maximum number of litters observed for any dam. Weighting by litter size ensures that larger litters contribute proportionally more to the estimate, reflecting their greater contribution to the next generation.

The mean litter interval $\bar{I}$ (mean time in years between consecutive litters of the same dam) for the population is computed from consecutive litter age pairs averaged across dams,

$$\bar{I}=\frac{1}{K-1}\sum_{k=1}^{K-1}\bar{a_{k+1}}-\bar{a_{k}}$$

and the mean number of litters per dam per year follows as $\bar{\ell }=\frac{1}{\bar{I}}$. This quantity requires at least some dams to have produced two or more litters; if all dams have only a single recorded litter, production projections are unavailable until more litter history accumulates.

### 2.8. Population Growth Projections

The Population Growth panel back-calculates the number of litters, active dams, sires, and replacement animals required to meet a user-specified annual production goal *G* (target progeny per year), given observed litter statistics and user-defined programme parameters. It is highly recommended for the user to specify a retirement date in the column for the dams as omitting this retirement date column causes dams to be counted as active beyond their actual breeding window, leading to systematically overoptimistic projections. A warning is displayed when this column is absent.

The number of litters needed per year, accounting for an expected litter correction rate *p_correction_*:

$$N_{\mathrm{litters}}=\left\lceil \frac{G}{\bar{\mathrm{mls}}\cdot \left(1-p_{\mathrm{correction}}\right)}\right\rceil$$

where $\bar{\mathrm{mls}}$ is the observed mean litter size and *p_correction_* ∈ [0,1] is the proportion of planned litters expected not to produce progeny. The litter correction rate can be set manually or estimated from historical data as the proportion of dam-years in which an active, non-retired dam did not produce a litter:

$$p_{\mathrm{correction}}=\max\left(0,\;\frac{1}{Y}\sum_{y=1}^{Y}\left(1-\frac{l_{y}^{\mathrm{obs}}}{l_{y}^{\exp}}\right)\right)$$

where $l_{y}^{\mathrm{obs}}$ is the number of litters observed in year *y*, $l_{y}^{\exp}=n_{y}^{\mathrm{active}}\times \bar{\ell }$ is the expected number based on active dams and mean litter rate in year *y*, and *Y* is the number of completed years with at least one expected litter. The floor at zero prevents negative correction rates when observed output exceeds expectation.

Similarly, the percentage of external sires can be set manually or derived from the population’s *N_e_*. Mills and Allendorf [29] recommended between 1 and 10 effective migrants per generation. If 10 is used to be on the safe side and expressed proportionally to the *Ne*, this implies an ext%_recommended_:

$$\mathrm{ext}{\%}_{\mathrm{recommended}}=\left\{\begin{array}{ll} >40\% & \mathrm{if}\;N_{e}<25\\ 40\% & \mathrm{if}\;25\leq N_{e}<50\\ 20\% & \mathrm{if}\;{50\leq N}_{e}<100\\ 10\% & \mathrm{if}\;100\leq N_{e}<200\\ 5\% & \mathrm{if}\;N_{e}\geq 200 \end{array}\right.$$

This recommendation tiers inversely with *N_e_* with smaller, more genetically vulnerable populations advised to import proportionally more unrelated genetic material to counteract inbreeding accumulation [27,28].

The split between litters from the own-population *N_litters_*_,*int*_ and external sire litters *N_litters_*_,*ext*_ follows from the external sire proportion *ext*%:

*N_litters_*_,*ext*_ = *round*(*N_litters_* ∙ *ext*%)

where values with a decimal below 0.5 are rounded down, and values with a decimal of 0.5 or above are rounded up. The number of litters from own-population sires is calculated as follows:

*N_litters_*_,*int*_ = *N_litters_* − *N_litters_*_,*ext*_

The number of active dams *N_dams_* required to produce *N_litters_* litters per year, given the mean number of litters per dam per year $\bar{\ell }$:

$$N_{\mathrm{dams}}\;=\;\left\lceil \frac{N_{\mathrm{litters}}\;}{\bar{\ell }}\right\rceil$$

The number of dams that must be recruited each year *N_dams_*_,*replaced*_ to maintain the active pool at *N_dams_*, given a user-specified maximum lifetime litter allowance per dam *m_dam_*:

$$N_{\mathrm{dams},\mathrm{replaced}}\;=\;\left\lceil \frac{N_{\mathrm{dams}}}{m_{\mathrm{dam}}\cdot \bar{\ell }}\right\rceil$$

The denominator $m_{\mathrm{dam}}\cdot \bar{\ell }$ is the expected number of years a dam remains active, so this expression gives the annual replacement rate needed to sustain the pool. The number of own-population sires *N_sires_*_, *selected*_ to be selected each year to cover planned internal litters, given a user-specified maximum lifetime litter allowance *m_sire_*:

$$N_{\mathrm{sires},\mathrm{selected}}\;=\;\left\lceil \frac{N_{\mathrm{litters},\mathrm{int}}}{m_{\mathrm{sire}}}\right\rceil$$

Unlike dams, a sire’s contribution is not necessarily limited to a fixed breeding window thanks to assisted reproduction techniques and semen storage. As such, *N*_*sires*,*selected*_ represents an annual selection target rather than a replacement rate in the strict sense.

The Dam Planner visualises each dam’s hypothetical breeding window as a Gantt chart. The start and end of the breeding window for dam *d* are defined as:

$${\mathrm{start}}_{d}=\;{\mathrm{DOB}}_{d}+\;{\tilde{a}}_{1}\;{\mathrm{end}}_{d}=\min({\mathrm{start}}_{d}+(m_{\mathrm{dam}}-{n_{d}}^{\mathrm{obs}})\;\cdot \;\bar{I},\;r_{d})$$

where *DOB_d_* is the date of birth of dam *d*, ${\tilde{a}}_{1}$ is the mean age at first litter, *m_dam_* is the maximum lifetime litter allowance, ${n_{d}}^{\mathrm{obs}}$ is the number of litters already produced by dam *d*, $\bar{I}$ is the mean litter interval, and *r_d_* is the retirement date for dam *d* specified in the data file. The *min*() operator ensures the window is truncated at the retirement date when one is provided.

The Progeny Planner compares expected versus actual progeny counts per year, where the expected count for year y is:

$$E[{\mathrm{progeny}}_{y}]\;=\;n_{\mathrm{active}\mathrm{dams},y}\;\cdot \bar{\ell }\;\cdot \;\bar{\mathrm{mls}}\;\cdot \;(1\;-\;p_{\mathrm{correction}})$$

and *n*_*active dams*,*y*_ is the number of dams whose hypothetical breeding window overlaps year *y*.

### 2.9. Mean Kinship

The mean kinship of the active breeding stock, in the Active Breeding Stock panel, is computed as the mean of all pairwise off-diagonal kinship values among breeders:

k¯breeders=1nb2∑i<jkij

where *n_b_* is the number of breeders and *k_ij_* is the kinship coefficient drawn from the full kinship matrix. This quantity is compared against the equivalent mean kinship computed for the entire own-population subset. Under random mating within the breeding group, ${\bar{k}}_{\mathrm{breeders}}$ equals the expected inbreeding coefficient of the next generation [30], making it a direct predictor of the genetic diversity of the next cohort.

Separately, numeric phenotype values in the breeder overview table are displayed using a delta representation $\delta i\;=\;\mathrm{xi}\;-\;{\bar{x}}_{\mathrm{population}}$, indicating each breeder’s deviation from the population mean.

### 2.10. Breeder Selection

In the Breeder Selection panel, mean kinship per candidate is computed as the average kinship to all own-population animals, and candidates are classified into tertiles derived from the full own-population kinship distribution following Oliehoek et al. [22]. Selection criteria for any phenotypic variable in the data file can be specified with a direction (≤, ≥, =, ≠), a target value, and a priority level. Mandatory criteria act as hard filters: candidates not meeting the criterion are excluded entirely. Preferred criteria act as soft filters: non-compliant candidates remain visible but are sorted to the bottom, ranked by the number of preferred criteria they fail. A birth year range slider restricts the candidate pool to a user-defined recruitment cohort. Numeric trait values in the candidate table are displayed in delta format (as defined above), indicating each candidate’s deviation from the population mean.

Criteria can be saved, which activates a Compliance Overview table showing, for each saved goal, the proportion of current breeders already meeting the criterion. Two summary statistics are reported: the mean mandatory compliance across all mandatory goals, and the number and percentage of current breeders passing all mandatory criteria simultaneously. Saved goals can additionally be imported into the Ranked Mating module as optional hard filters.

### 2.11. Ranked Mating

The Ranked Mating module ranks all breeding-stock animals of the opposite sex relative to a selected test parent. The expected inbreeding coefficient (indicated as ‘COI’ in the application) of prospective offspring is computed directly as $F_{i}=\;k_{s_{i},\;d_{i}}$ (see Inbreeding and pairwise kinship coefficients section), from the stored kinship matrix. Maximum COI increase over the parental average, defined here as δ*F*, distinct from both the individual lifetime increase *ΔF*_i_ and the population-level rate $\bar{\Delta F}$ defined above, is applied as a hard filter:

$$\delta F=F_{i}-\frac{F_{d_{i}}+F_{s_{i}}}{2}\leq \delta_{\max}$$

Filtering on δ*F* rather than on *F_i_* is a deliberate design choice. Individual *F_i_* is largely a function of pedigree depth: deeply recorded pedigrees yield higher *F_i_* values even under careful mating, while shallow pedigrees can produce low *F_i_* values even under poor mating choices. The increase δ*F* isolates the contribution of the specific mating from accumulated genealogical history and is the quantity directly comparable to the population-level rate of inbreeding ($\bar{\Delta F}$) used elsewhere in the application; it is also the quantity over which the breeding manager exerts immediate control.

As a suggested default, δ_max_ is set to 0.01, aligned with the 1% per-generation inbreeding threshold corresponding to *N_e_* < 50 applied throughout the application. This provides a practical per-mating ceiling that prevents individual matings from contributing disproportionately to inbreeding accumulation. After filtering, candidates are ranked in ascending order of δ*F*, so the matings contributing least to inbreeding accumulation appear first. User-defined phenotypic criteria can be applied as additional hard filters. If the constraint yields no eligible candidates, δ_max_ can be incrementally relaxed or phenotypic criteria reviewed, prioritising the candidate that minimises the deviation from the 0.01 threshold. This constraint limits the rate at which inbreeding accumulates across generations, rather than targeting the absolute inbreeding level of individual offspring.

The module also displays each candidate’s mean kinship to the population, colour-coded by tertile (green: bottom third; orange: middle third; red: top third), matching the Breeder Selection panel. This allows the user to identify overrepresented candidates.

### 2.12. Validation of Genetic Metrics Using R Packages

To verify the correctness of RAPPORT’s genetic computations, inbreeding coefficients, kinship values, equivalent complete generations, rate of inbreeding, and effective population size were independently recomputed from the same test-case pedigree and data files using the R packages pedigree (v1.4.2; for inbreeding via Henderson’s tabular method) and kinship2 (v1.9.6.2; for kinship via the kinship matrix diagonal). Both packages received the same cleaned pedigree (single parents set to unknown, founder insertion, topological sorting) to ensure identical input. Inbreeding coefficients were compared element-wise between all three implementations. ECG, ΔF, and Ne were recomputed over the own-population subset, matching the reference population filtering applied by RAPPORT, using the Gutiérrez et al. [26] individual increase in inbreeding method. The validation script is available alongside the application source code.

## 3. Test Case: A Belgian Assistance Dog Breeding Program

### 3.1. Dataset

The RAPPORT application was evaluated using anonymized data from a Belgian assistance dog breeding program. The data file contained 421 animals, with 242 males and 179 females, covering birth years 2013–2026. For each animal, the data file included date of birth (‘Date of Birth’); sex (‘Sex’); active breeding stock status (‘Breeder’); population membership (‘Own Population’); retirement date (‘Retirement Date’, set two months after the birth of their last litter for retired dams); and the following health phenotypes: hip joint laxity index (‘Hip_LI’), FCI (Fédération Cynologique Internationale) hip dysplasia grade (‘Hip_FCI’) and elbow fragmented medial coronoid process status (‘FMCP’). The raw pedigree file contained 852 entries, with 37 where both parents were unknown, 10 with only the sire known and 1 with only the dam known.

### 3.2. Data Input and Preprocessing

**Figure 2:**
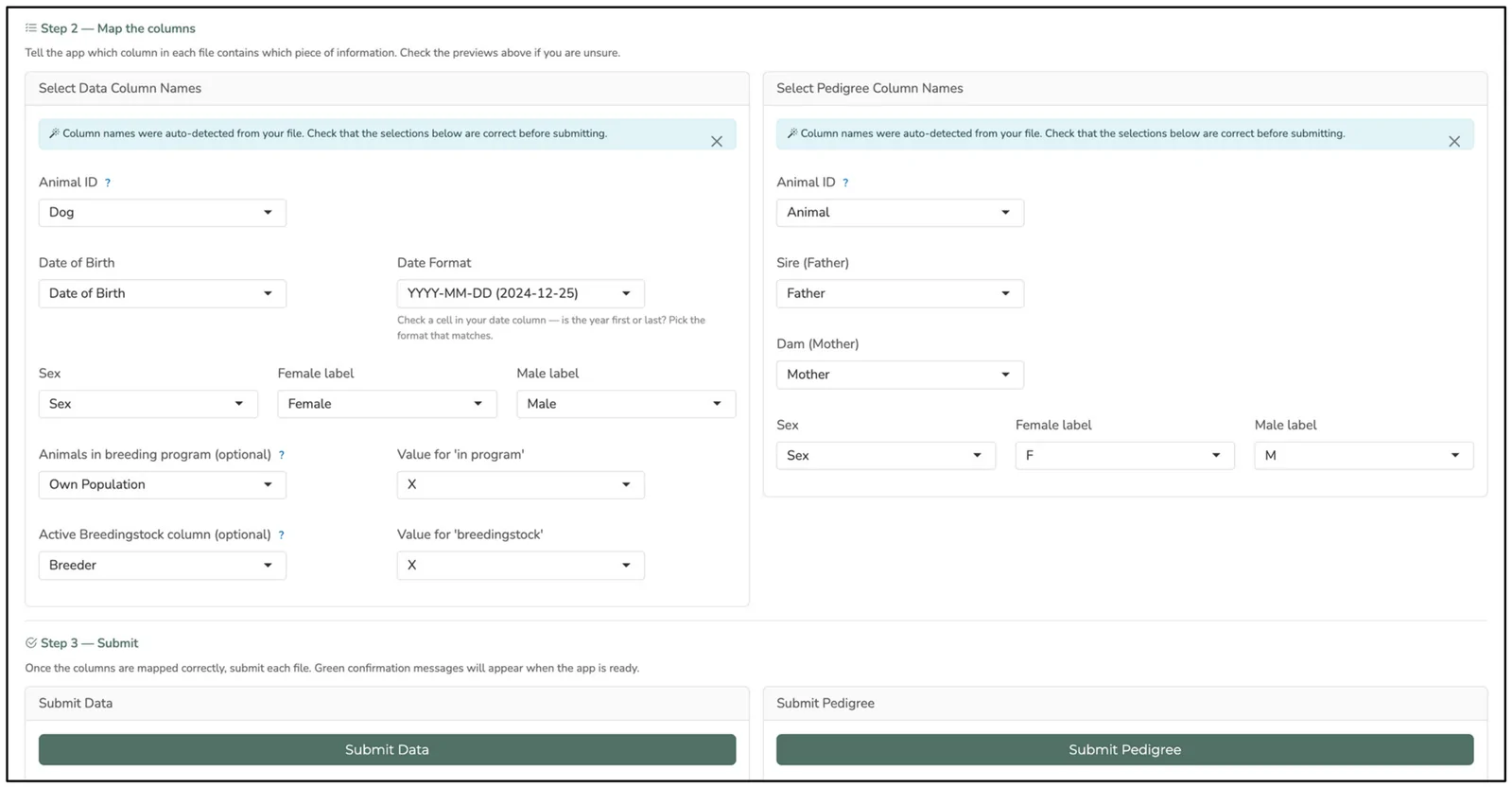
The ‘Data Input’ panel after data upload and column auto-detection. Left: data file column mappings with auto-detected fields; the date format still shows the default and must be set manually. Right: pedigree file column mappings. Blue banners prompt the user to verify all auto-detected selections before submitting.

On upload, RAPPORT’s column auto-detection correctly pre-selected the column mappings for both files; the user only needed to specify the correct date format for the date of birth column (‘DD/MM/YYYY’) (Figure 2). On pedigree submission, founder rows were added for those individuals that were included as parents but were not mentioned in the first column themselves, and the pedigree was topologically sorted. The quality check report flagged 3 dams that were recorded as males; the user was warned that their sex had been automatically corrected to female, and that if it was the parentage that was swapped instead of the sex, the user should change this manually in the source file itself and re-upload. All animals in the data file were present in the pedigree.

### 3.3. Population

The ‘Population Overview’ tab summarized the own-population subset as 377 animals across 65 litters, with a mean litter size of 6 (95% CI: 5.3–6.6), a generation interval of 3.57 years, with an ECG of 6.4, and an *N_e_* estimated at 232 (95% CI: 205–259), placing the population in the ‘*N_e_* adequate’ category ($\bar{\Delta F}\;$= 0.0022, 95% CI: 0.002–0.0024), with no immediate intervention needed. The ‘Birth Statistics by Year’ provides a visual overview of the population through an interactive bar chart with a selectable metric (e.g., mean litter size, number of progeny born), accompanied by a yearly summary table. Clicking a bar in the chart displays a detailed table of the individual animals born in the selected year.

The ‘Litters’, ‘Dams’, and ‘Sires’ tabs provided similar overviews of the number of litters, dams, and sires per year, each with the option to click to display the corresponding subset. Individual animal records could be accessed via the ‘Animal Info’ tab; for example, LAB_0023 had a COI of 0.011, seven littermates, and parents LAB_0665 and LAB_0014, with an ‘A’ FCI hip score and an unaffected FMCP status.

**Figure 3:**
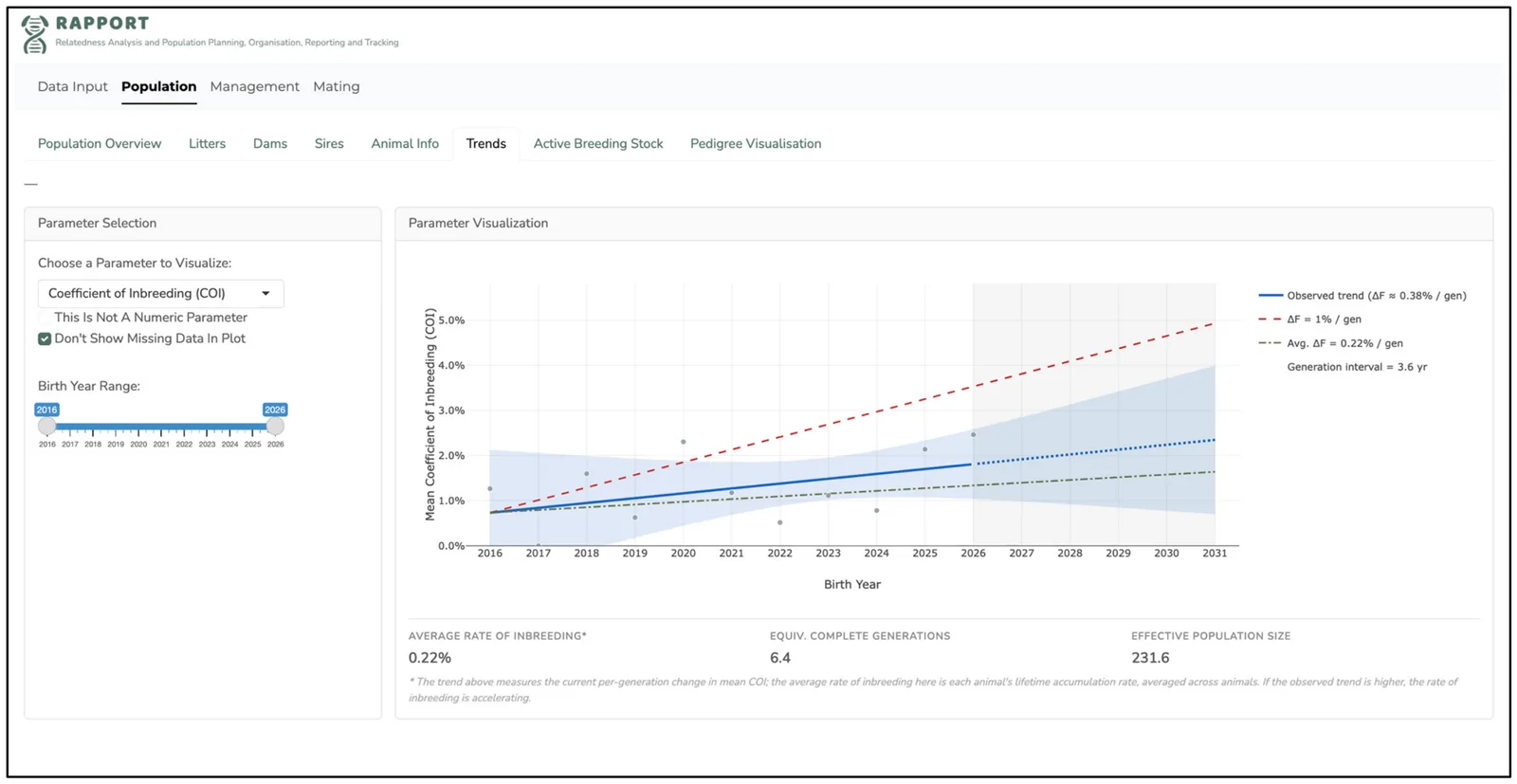
Trends panel showing mean coefficient of inbreeding (COI) per birth year with weighted linear regression (solid blue line), 95% confidence band (blue shaded area), and five-year forward projection. The dashed red line marks the ΔF = 1% per generation benchmark, converted to a per-year slope using the estimated generation interval of 3.6 years. The green dotted line shows the average rate of inbreeding (0.22% per generation). Summary statistics are reported below the plot.

The Trends tab visualized the mean COI per birth year alongside a weighted linear regression projected five years forward with a 95% confidence band. A dashed reference line corresponding to *ΔF* = 1% per generation (converted to a per-year slope using the estimated generation interval of 3.6 years) served as a management benchmark. The observed trend (≈0.38% per generation) remained well below this threshold. The divergence between the observed trend and the average rate of inbreeding $\bar{\Delta F}$ (0.22%) indicated that inbreeding has been accelerating over time (Figure 3).

As for the phenotypes, hip joint laxity showed a decreasing trend, and FCI grade A was the most prevalent hip score, accounting for 82.8% of records. Roughly 14% of animals born in 2024 were recorded as affected with FMCP.

The active breeding stock comprised 23 dams and 41 sires, of which 49 originated from the own population, with one external dam and 14 external sires. Breeders had a mean pairwise kinship of ${\bar{k}}_{\mathrm{breeders}}$ = 0.0505, against ${\bar{k}}_{\mathrm{population}}$ = 0.0538 for the own-population subset. Active breeding animals showed mostly favourable phenotypes compared to the overall population, with a lower mean hip laxity index (median 0.3 vs. 0.33), a higher proportion of FCI hip grades A and B (100% vs. 97.5%), and lower FMCP prevalence (0% vs. 10.7%).

The Pedigree Visualisation tab offered two views: a full pedigree of the reference population, and an individual pedigree for any selected animal tracing back four generations with common ancestors highlighted in red.

### 3.4. Management

The Population Growth panel was configured with a target of 150 puppies per year, a maximum of 4 litters per dam, 3 per sire and the litter correction rate was manually set to 5%. Based on the current *N_e_*, the recommendation is 5% of litters to originate from external sires. From these inputs, the panel calculated a requirement of 27 litters per year (24 from own-population sires, 3 from external sires), 25 active dams, with 6 dams and 8 own-population sires to be selected per year. The forward projection for the current year, applying the 5% correction rate to 19 currently active dams, stands at 119 puppies (95% CI: 106–132), falling short of the production goal by 31 puppies, meaning 6 more litters or 5 more dams are required to reach the goal. Last year, 82% of the expected number of puppies was born (70 born versus 85 expected).

The Dam Planner showed 19 dams currently within their hypothetical breeding window, 5 dams that had not yet produced a first litter, 3 due for their final litter, and 11 dams retired. The mean litter interval was 0.9 years, giving a mean of 1.1 litters per year per dam and dams had their first litter on average at the age of 2.3 years.

The Progeny Planner tab displayed a historical comparison of expected versus actual progeny counts per year, with coverage last year at 82%. Clicking a year bar revealed which dams produced litters that year and which were expected to but did not.

**Figure 4:**
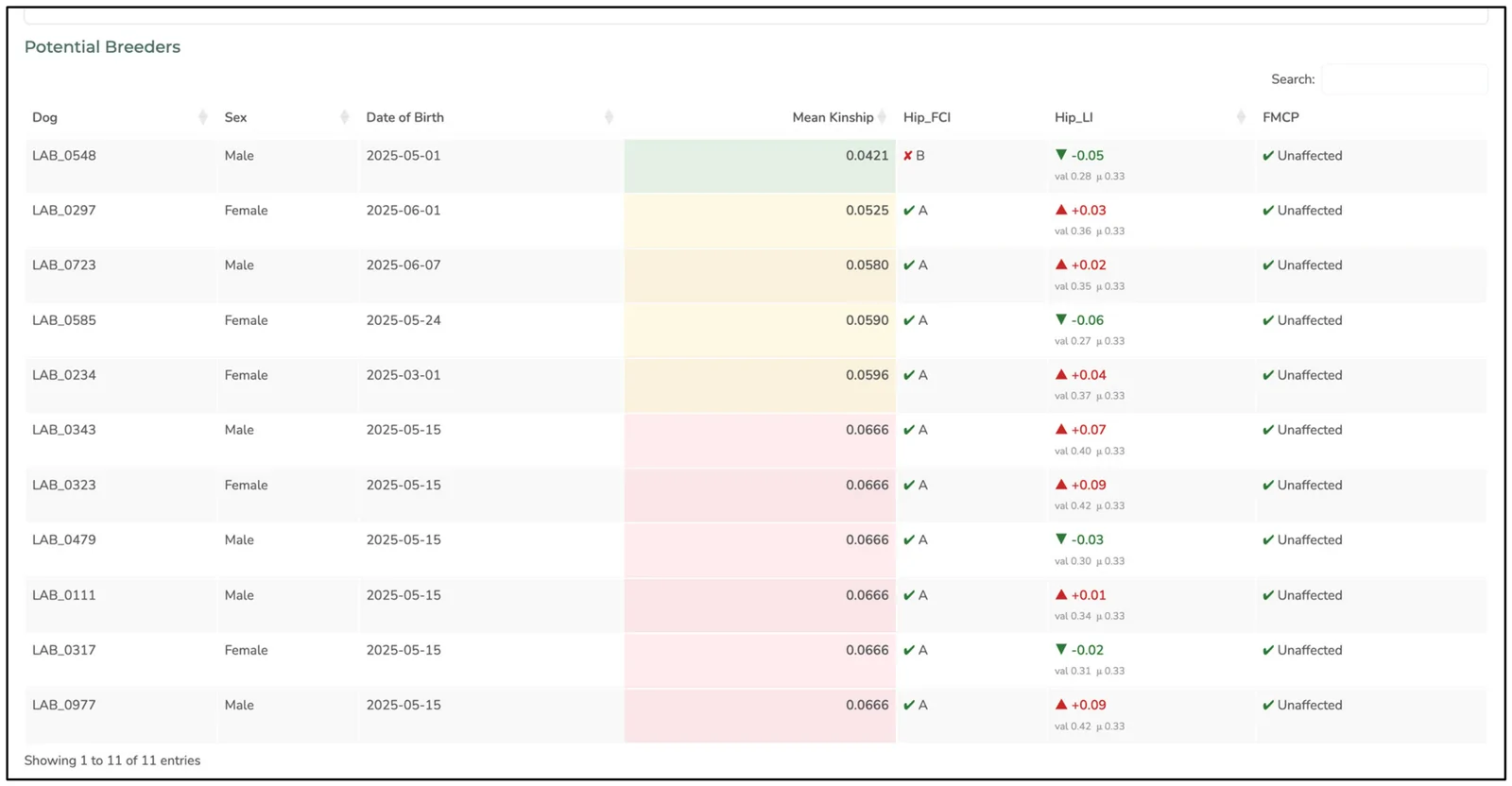
Breeder Selection panel showing the filtered Potential Breeders table, sorted by ascending mean kinship. Candidates are colour-coded by population-wide mean kinship tertile: green (low, prioritise), orange (middle, acceptable), and red (high, use cautiously). Phenotypic variables are displayed in delta format, showing each candidate’s deviation from the population mean with directional arrows. Check marks and crosses indicate pass/fail status for health screening results, respectively.

The Breeder Selection panel indicated that 6 dams and 8 sires needed to be recruited from the animals born in the previous year, of which 2 dams and 2 sires had already been selected. Selection criteria were set to a hip laxity index ≤ 0.45 and elbows free of FMCP as mandatory criteria, as well as an FCI hip grade set to ‘A’ as a preferred criterion. The birth year range was set to 1 March 2025 through 1 July 2025, targeting animals at approximately one year of age, covering 41 animals. Of these, 11 eligible candidates (5 potential dams, 6 potential sires) were returned, sorted by ascending mean kinship (Figure 4). Tertile boundaries for mean kinship were 0.0465 and 0.0608; one candidate fell in the low (green) tertile, four in the middle (orange), and six in the high (red). A future sire from the low tertile and a future bitch from the middle tertile with the most favourable phenotype scores were selected and converted to breeders within the app. The Compliance Overview showed that 92% of current active breeders met all mandatory criteria, with five breeders lacking hip joint laxity records and therefore not passing compliance.

### 3.5. Mating

To illustrate mate selection, female LAB_0235 (*F* = 0.0159) was selected as the test parent in the Mating tab. Applying a maximum COI increase of 0.01 over the parental average led to 14 eligible candidates. These were ranked in the Ranked Candidates table so that the genetically safest combinations appeared first; the top-ranked candidate produced an expected *δF* of −0.0303, meaning the combination is expected to produce offspring less inbred than the parental average. Importing saved mandatory health goals from the Breeder Selection module as additional hard filters further reduced the eligible pool to 11 candidates.

### 3.6. Validation of Genetic Metrics Using R Packages

Inbreeding coefficients computed by RAPPORT, the pedigree package, and kinship2 were numerically identical across all animals in the cleaned pedigree. When ECG, ΔF, and Ne were recomputed over the own-population subset (*n* = 377), the validation script returned a mean ECG of 6.44, ΔF of 0.0022, and Ne of 231.6, consistent with the values reported by RAPPORT.

## 4. Discussion

No currently available tool integrates genetic monitoring with the daily operational management of a breeding program in a single accessible interface. Tools such as Retriever and Pointer [15] and optiSel [14] offer great methodological depth for genetic analysis, while the IWDR [11,20,21] represents a very elaborate integrated platform, combining EBV-based genetic selection, inbreeding management, phenotypic record-keeping, and health monitoring across collaborating organizations. RAPPORT does not aim to compete with these, but rather aims to focus on those aspects which are less represented in the currently available tools (see Table 1). In more detail, the aim is to bring genetic monitoring into the daily running of a program—production planning, dam scheduling, breeder recruitment, and mate selection—in a single interface accessible to users without programming skills or quantitative genetics training. Designed for the manager of a breeding program to make these decisions from their own data without external infrastructure, it is a web application developed in R using the Shiny framework, building on R’s established infrastructure for statistical and genetic analysis. Users upload two Excel files, a pedigree and a data file, and map their column names interactively. The application then computes inbreeding coefficients and pairwise kinship, estimates effective population size *N_e_* and rate of inbreeding *ΔF*, and generates an integrated management environment that covers population monitoring, production planning and projections, phenotype-integrated breeder selection, and mating evaluations. All outputs are presented with plain-language explanations and contextual guidance, and no module requires the user to interpret raw statistical output.

The practical test case using data from a Belgian assistance dog program illustrated what the application can offer and the conditions under which it is most informative. With a pedigree of 852 entries spanning multiple generations, genetic metrics were based on a reasonably complete record, and several years of litter history in the data file supported correct management projections. As such, RAPPORT fulfils the criteria as stated in Table 1. In more detail, a current production shortfall was identified, while at the same time a diversity context was provided. As the *N_e_* was considered adequate, the focus can be put on increasing the number of animals and the management panels quantified exactly how many additional dams and litters were needed to meet the production goal. The breeder selection panel returned a ranked candidate list with kinship framing and phenotypic filters applied simultaneously, and the mating evaluation narrowed 14 eligible sires to 11 after applying inbreeding threshold and health criteria. These are decisions that many program managers often make by manually cross-referencing individual records, and without any quantitative support at all.

While this is not specific to RAPPORT, a fundamental limitation is pedigree completeness. Inbreeding coefficients computed from Henderson’s tabular method are conditional on pedigree depth: unknown parents are treated as unrelated founders, which means *F* values are underestimated wherever ancestry is missing. This leads to a bias in the estimation of $\bar{\Delta F}$ and therefore *N_e_*: incomplete pedigrees produce optimistic *N_e_* estimates, in a direction least helpful to the manager of an at-risk population. To avoid further overestimation, founders were excluded here from the $\bar{\Delta F}$ calculation (animals with no known ancestry and therefore no inbreeding information), which pulls *N_e_* downward relative to including them. This partially counteracts the potential upward bias introduced by pedigree incompleteness. The ECG statistic provides a useful indicator of pedigree depth, but it cannot correct an *N_e_* or $\bar{\Delta F}$ bias. It can however give an idea of the trustworthiness of the latter two. We deliberately chose not to impose a minimum ECG threshold for inclusion in calculations. In semi-closed breeding programs, the animals most likely to have shallow pedigrees are those introduced from external sources, whose genetic contribution is precisely what these programs rely on to manage inbreeding. Excluding these animals would risk discouraging the importation of unrelated genetic material. Programs with shallow registration histories or incomplete founder records should interpret *N_e_* estimates and inbreeding trends with caution. Other tools developed specifically for *N_e_* estimation in pedigreed populations, including Retriever [15] and POPREP [16], provide dedicated diagnostic environments and may be more suitable where *N_e_* estimation is the primary analytical goal. RAPPORT’s *N_e_* estimate is a contextual indicator, meant to support management recommendations, not a precise demographic parameter. For example, the recommended external sire percentages are practical starting points based on Mills and Allendorf [29]. However, in domesticated breed populations, imported sires can share considerable background relatedness with the population, reducing the effective contribution per migrant compared to the unrelated external populations assumed by Wright’s island model. Additionally, in assistance dog breeding programs, imported sires and their progeny are subject to stringent health and behavioral selection criteria, meaning that a substantial fraction of imported genetic material may not propagate into the next breeding generations. The proposed percentages are therefore set above the theoretical recommendations and should also be adapted to program-specific constraints on sire availability and inter-program collaboration. While periods of intense selection, popular sire effects, or abrupt changes in program size will be averaged over in the single summary figure, the annual mean inbreeding trend visualized in the ‘Trends’ tab is a complementary diagnostic here.

The reliability of the demographic projections in the Management module depends on the completeness and correctness of the provide data file. All production calculations are derived from the observed litter statistics of the own-population subset, meaning that programs with short data histories (fewer than two or three years of litter records, or with all dams having produced only a single litter) cannot compute a reliable mean litter interval and therefore cannot generate reliable projections. The app flags this situation, but for a recently established program, users should be aware that planning projections gain reliability as litter history accumulates. Additionally, omitting the optional retirement date column causes dams that have left the active breeding stock to continue being counted as active, systematically inflating expected production. This is also flagged with a warning.

To validate RAPPORT’s core pedigree-based metrics, inbreeding coefficients and kinship values were recomputed from the test-case pedigree using the pedigree and kinship2 R packages. Results were numerically identical, as expected given that the underlying algorithms are the same. RAPPORT reports Ne and ΔF on a per-generation basis using equivalent complete generations, which does not require dates of birth. Where dates of birth are available for the reference population, the Trends module converts the per-generation rate to a calendar-year scale using the estimated generation interval. Calendar-based trend tools such as POPREP require complete birth date records and are therefore not directly comparable. This comparison constituted a baseline consistency check of the pedigree-based genetic metrics, not a validation of the software framework as a whole.

Finally, there are some specific limitations we would like to address for RAPPORT. The operational management features (dam scheduling, production projections, litter statistics) are designed around a dam-offspring breeding model that applies to any species with individually identifiable dams and discrete breeding events, including species producing single offspring, and have been tested with a canine breeding program. While the underlying genetic methodology is applicable across species, the management modules may require adaptation for species with different reproductive biology (e.g., avian or aquatic species). Additionally, as discussed above, all genetic metrics computed by RAPPORT are conditional on pedigree completeness and depth, and users of programs with shallow registration histories should interpret results accordingly. Furthermore, RAPPORT does not compute EBVs internally. However, externally calculated EBVs can be imported as numeric trait columns in the data file. They are then available throughout the application as any other phenotypic variable: visualized in the population trends, applied as selection criteria in the Breeder Selection panel, or as hard filters in Ranked Mating. Finally, a potential limitation is that the kinship matrix scales as n^2^ with n = the number of individuals, meaning that memory use and computation time grow rapidly with pedigree size. The application is designed for programs producing up to a few hundred offspring per year; pedigrees accumulating to thousands of individuals are likely to encounter performance limitations. As a reference, on a mid-range laptop (MacBook Air, Apple M2, 24 GB RAM), the kinship matrix for the test pedigree (852 entries) was computed in approximately 200 ms.

Looking ahead, a valuable analytical extension would be the decomposition of inbreeding into “new” (arising from recent common ancestors) and “ancestral” (inherited from more distant common ancestors) components, as proposed by Kalinowski et al. [31]. This distinction would help users assess whether current management decisions are contributing to inbreeding accumulation beyond what is inherited from the historical pedigree structure. A further natural extension would be the incorporation of genomic data. Even though it has been suggested that in some cases a sufficiently complete pedigree is still the preferred tool to control the rate of inbreeding [32], integrating SNV-based kinship estimates would remove the pedigree completeness dependency entirely and may substantially improve the reliability of estimation and breeder selection in programs where genomic data are available and pedigree data is limited.

## 5. Conclusions

RAPPORT integrates pedigree-based genetic monitoring with the operational management of breeding programs in a single accessible interface. It is most informative in programs with well-maintained pedigrees and sufficient litter history; where these are lacking, outputs should be interpreted with caution. It is not a substitute for specialist expertise or more powerful platforms where these are accessible, but for the many programs that operate without either, it offers a practical starting point for more informed breeding decisions.

## Data Availability

The original data presented in the study are openly available in GitHub at GitHub-BirgitDeboutte/RAPPORT: R Shiny application for breeding program management.

## Abbreviations

The following abbreviations are used in this manuscript:

BCAS	Breeding Colony Analytics Software
CI	Confidence Interval
COI	Coefficient of Inbreeding
DOB	Date of Birth
EBV	Estimated Breeding Value
ECG	Equivalent Complete Generations
FCI	Fédération Cynologique Internationale
FMCP	Fragmented Medial Coronoid Process
GPL	General Public License
IBD	Identical by Descent
IWDR	International Working Dog Registry
MoBPS	Modular Breeding Program Simulator
N_e_	Effective Population Size
RAPPORT	Relatedness Analysis and Population Planning, Organisation, Reporting and Tracking
SNV	Single Nucleotide Variant
ΔF	Rate of Inbreeding
δF	Per-mating Inbreeding Increase over Parental Average
